# Prediction of heavy-section ductile iron fracture toughness based on machine learning

**DOI:** 10.1038/s41598-024-55089-3

**Published:** 2024-02-26

**Authors:** Liang Song, Hongcheng Zhang, Junxing Zhang, Hai Guo

**Affiliations:** 1 School of Intelligent Manufacturing and Automotive Engineering, Luzhou Vocational & Technical College, Luzhou, 646000 China; 2 College of Computer Science and Engineering, Dalian Minzu University, Dalian, 116600 China

**Keywords:** Materials science, Structural materials

## Abstract

The preparation process and composition design of heavy-section ductile iron are the key factors affecting its fracture toughness. These factors are challenging to address due to the long casting cycle, high cost and complex influencing factors of this type of iron. In this paper, 18 cubic physical simulation test blocks with 400 mm wall thickness were prepared by adjusting the C, Si and Mn contents in heavy-section ductile iron using a homemade physical simulation casting system. Four locations with different cooling rates were selected for each specimen, and 72 specimens with different compositions and cooling times of the heavy-section ductile iron were prepared. Six machine learning-based heavy-section ductile iron fracture toughness predictive models were constructed based on measured data with the C content, Si content, Mn content and cooling rate as input data and the fracture toughness as the output data. The experimental results showed that the constructed bagging model has high accuracy in predicting the fracture toughness of heavy-section ductile iron, with a coefficient of coefficient (*R*^2^) of 0.9990 and a root mean square error (RMSE) of 0.2373.

## Introduction

### Performance design of heavy-section ductile iron

Heavy-section ductile iron is a widely used cast iron material. Due to its good fracture toughness, tensile strength, plasticity and antiradiation properties, it is commonly used in harshly demanding fields such as nuclear power, wind power, storage and transportation of nuclear spent fuel and high-speed railroads^1–3^. In view of the increasingly complex working conditions of heavy-section ductile iron, its safety is the most important consideration in engineering applications, and fracture toughness is an important basis for safety assessment^4^. At present, only German, Japanese, and individual scholars have carried out fracture toughness mechanism research on nuclear spent fuel storage and transportation containers with a wall thickness of less than 150 mm^5–7^. Panneerselvam^8^ studied the low-temperature fracture toughness of heavy-section ductile iron containers with 150 mm wall thickness, and the results showed that an increase in pearlite content led to a decrease in fracture toughness. Singh^9^ found that the fracture surface of heavy-section ductile iron has a special metal oxide film, which greatly reduced the fracture toughness and elongation of the material. The film was observed was due to the long cooling time, which is obviously different from ordinary ductile iron. Song and Guo^10,11^ studied the influence of silicon content and nodulizers on heavy-section ductile cast iron, and found that lower silicon content can reduce the performance of chunky graphite iron, especially in its capacity to demonstrate good impact performance at low temperatures.

At present, research on the fracture toughness mechanism of heavy-section ductile iron is relatively limited, and the chemical compositions of the main components of heavy-section ductile iron influence and interfere with each other, resulting in high preparation costs and long design cycles, and systematic research on prediction of heavy-section ductile iron fracture toughness is scarce, which seriously affects the practical application of heavy-section ductile iron.

### Application of machine learning to material design

Turing Award winner James Gray classifies scientific research into four types of paradigms: experimental induction, model derivation, simulation and data-intensive scientific discovery. As part of the fourth paradigm of scientific research, ML is a multidisciplinary field that covers numerous disciplines. Machine learning is mainly the study of how computers mimic human learning behavior, acquire new knowledge or experience, and reorganize existing knowledge structures to improve their performance, and ML is also widely used in other fields of scientific research^12^.

Machine learning has been widely used in materials design. Chiniforush^13^ achieved phase prediction of novel high-entropy alloys based on the random forest classifier model. Anand^12^ made a breakthrough in topological feature engineering for perovskite material design by using a machine learning model. Huo^14^ utilized a semisupervised machine learning approach to unlock a large amount of inorganic materials in literature synthesis information and processed it into a standardized machine-readable database. Schmidt^15^ used a machine learning model to screen out materials with high catalytic activity and predicted and optimized the catalytic efficiency of the materials by establishing a relationship between the material surface structure and the rate of catalysis reaction. However, the prediction of the fracture toughness of heavy-section ductile iron has not been reported.

### Structure of this thesis

The solidification time of large heavy-section ductile iron castings is too long, leading to defects such as spheroidal fading, graphite distortion and graphite floating, which have a greater impact on the morphology of graphite and the mechanical properties and microstructure of ductile iron^16^. To obtain high toughness heavy-section ductile iron castings to meet mechanical properties demands of nuclear power, wind power and other fields, it is necessary to effectively control the matrix organization of heavy-section ductile iron castings. Graphite must be refined to improve the roundness of graphite balls and reduce the formation of deformed graphite. By adjusting the content of alloying elements or adjusting the cooling rate to adjust the matrix organization and graphite morphology of heavy-section ductile iron, heavy-section ductile iron castings with high performance can be obtained^17^. However, preparing heavy-section ductile iron is costly and time-consuming, and the chemical compositions of its main constituents interact with each other, which seriously restricts the application of high fracture toughness heavy-section ductile iron^18,19^. In the preliminary work, the project team and Qiqihar No.1 Machine Tool Factory have prepared 20 BQH-20 spent fuel transport containers, and A series of preparation process articles were published, the National Natural Science Foundation for large nuclear spent fuel storage and transportation container was applied^20–22^. The purpose of this study is to verify the application of the developed prediction model to the actual production of heavy-ductile iron products. This paper adopts a physical simulation method and homemade physical simulation casting system. By adjusting the contents of C, Si, and Mn in heavy-section ductile iron, 18 cubic physical simulation test blocks with a wall thickness of 400 mm were prepared, and each specimen was selected from the edge to the core of the test block. Seventy-two specimens with different compositions and cooling times of heavy-section ductile iron were prepared, and the effects of C, Si, Mn and cooling speed on the graphite morphology, microstructure and fracture toughness of heavy-section ductile iron were studied. The measured data were standardized to ensure that the prediction model error was small. According to the previous basis of the project team, the prediction of dielectric loss of polyimide nanocomposite films was constructed by using machine learning models such as support vector machine regression, multi-layer perceptron regression, bagging and random forest, and good expected results were achieved^23^. Therefore, this paper constructs machine learning models such as XGBoost, SVR, MLP regression, Gaussian process regression, bagging and random forest. The model is trained and verified by cross-validation technology, and its parameters are optimized by genetic algorithm to obtain high-precision fracture toughness prediction model of heavy section ductile iron.

### Effect of different factors on the fracture toughness of heavy-section ductile iron

In actual production, the five major elements for the preparation of heavy-section ductile iron are mainly carbon (C), silicon (Si), manganese (Mn), sulfur (S), and phosphorus (P). These elements and cooling rate have an important influence on the properties of heavy-section ductile iron^24–29^. The following is also the basis for the selection of chemical composition range for the preparation of samples in this paper.

### Effect of C

C is the basic element of heavy-section ductile iron and helps in graphitization, and the general content of Carbon is controlled between 3.5 and 3.9 wt%. Too high carbon content leads to graphite floating, too low leads to low spheroidization rate and reduced mechanical properties.

### Effect of Si

Si is strong graphitization element, which can effectively reduce the white iron tendency, increase the amount of ferrite, and improve the roundness of graphite ball. However, silicon will also increase the ductile–brittle transition temperature and reduce the impact toughness. Therefore, the silicon content should not be too high, generally controlled between 1.7 and 3.8 wt%.

### Effect of Mn

The main role of Mn is to increase the stability of pearlite and promote the formation of carbides, these carbides segregate at grain boundaries, which greatly reduces the toughness of ductile iron. The preparation of heavy-section ductile iron generally requires that it has a ferrite matrix to obtain high fracture toughness. However, due to the long cooling time of the core, it is easy to generate a large amount of pearlite and affect its toughness. Generally, the Mn content is limited to less than 0.5 wt%.

### Effect of S and P

In the preparation of heavy-section ductile iron, the content of S and P is often controlled as low as possible. Excessive S content will lead to hot brittleness and excessive P content will lead to cold brittleness of ductile iron.

### Effect of cooling rate

For heavy-section ductile iron, due to the increased wall thickness, casting solidification is slow, spheroidal fading, coarse graphite, chunky graphite, elemental segregation and other defects easily occur, and the microstructure and fracture toughness of heavy-section ductile iron are significantly different from those of normal ductile iron. To obtain high fracture toughness heavy-section ductile iron, water cooling is generally used to increase the cooling solidification rate. However, because the actual production of the heavy-section ductile iron wall thickness is different, even with the use of water cooling, the solidification time of the core test block is often more than 150 min, and the method cannot unlimitedly reduce its cooling rate to increase its fracture toughness.

### The relationship between micro-structure and four factors

**Figure 1:**
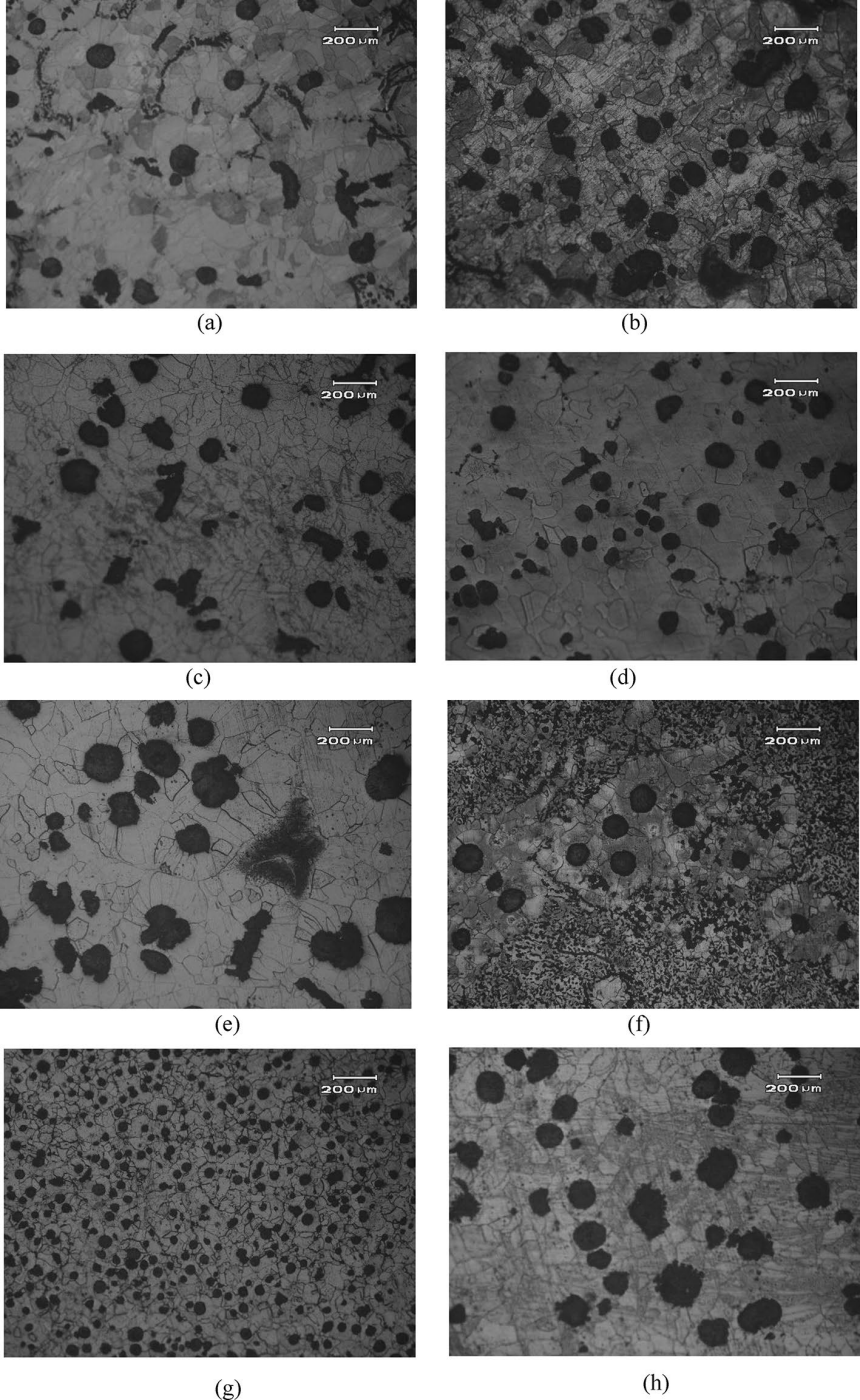
Effect of C, Si, Mn and cooling time on micro-structure of heavy-section ductile iron (part of the measured data) **(a)** C contents is 3.3 wt%, **(b)** C contents is 3.6 wt%,** (c)** Si contents is 1.9 wt%,** (d)** Si contents is 2.3 wt%,** (e)** Mn contents is 0.1 wt%,** (f)** Mn contents is 0.7 wt%,** (g)** Cooling time (min) 145, **(h)** Cooling time (min) 265.

In order to explore the relationship between the four factors (C content, Si content, Mn content, cooling rate), fracture toughness and the microstructure, samples with different (C content, Si content, Mn content, cooling rate) were selected for research, as shown in Fig. 1. Figure 1 shows the effects of C, Si, Mn and cooling rate (part of the measured data is selected) on the microstructure of heavy-section ductile iron. Figure 1a,b have different C contents, which are 3.3 wt% and 3.6 wt%, respectively, and the other components are the same. Figure 1c,d have different Si contents of 1.9 wt% and 2.3 wt%, respectively, and the other components are the same. Figure 1e,f have different Mn contents, which are 0.1 wt% and 0.7 wt%, respectively, and the other components are the same. Figure 1g,h have different cooling rates, 145 min and 265 min, respectively, and the other components are the same.

It can be seen from Fig. 1a,b that an appropriate increase in C content can increase the promotion of graphite nucleation and increase the number of graphite spheres. When the Si content reaches 2.3 wt%, the number of graphite spheres increases and the roundness increases, as shown in Fig. 1c,d. The increase of Mn content leads to a significant increase in the number of pearlite in the matrix structure, and a large amount of chunky graphite is produced, as shown in Fig. 1e,f. When the cooling rate of heavy section ductile iron is accelerated, the number of graphite balls is greatly increased and the graphite morphology is improved, as shown in Fig. 1g,h.

**Figure 2:**
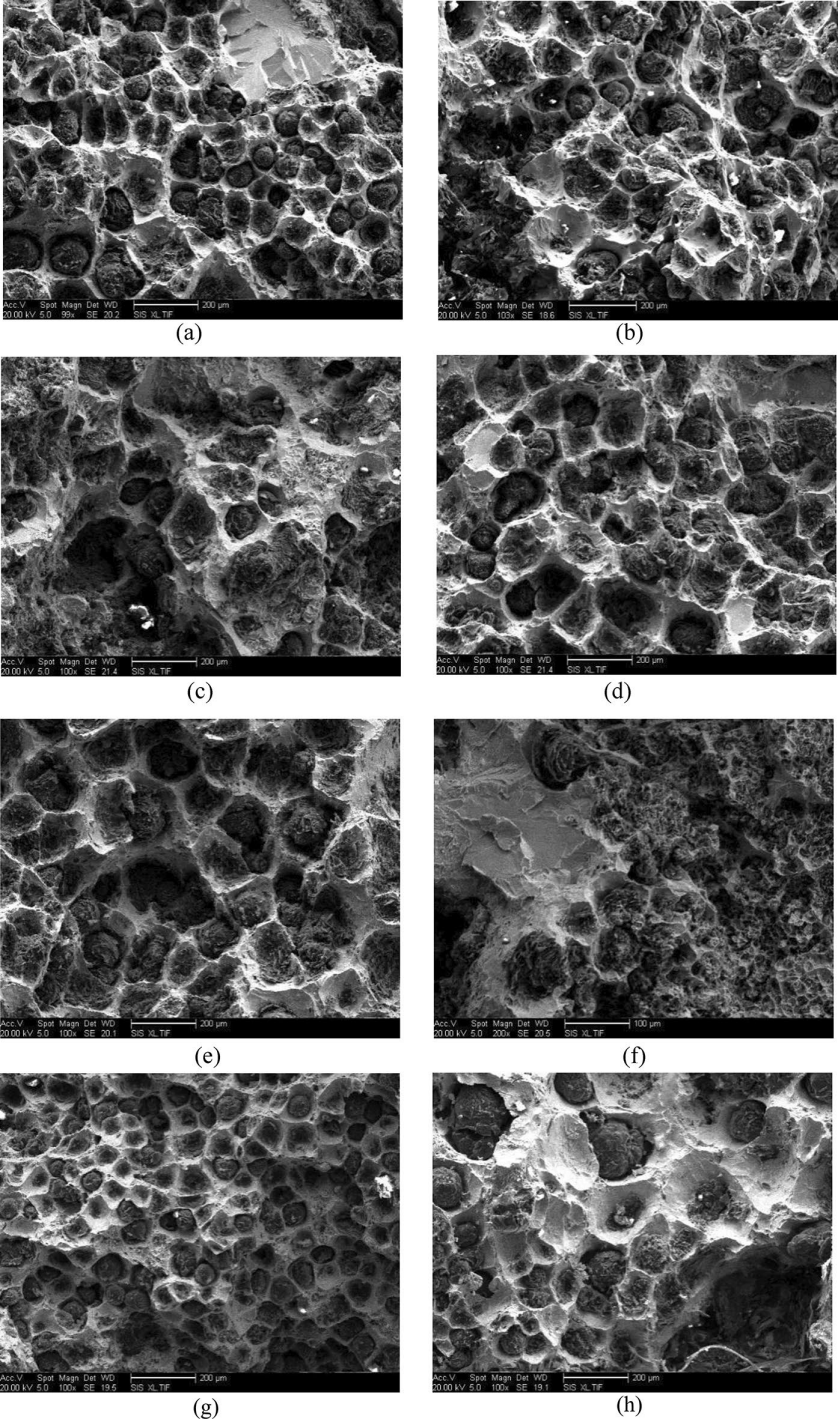
The fracture toughness fracture morphology of heavy-section ductile iron **(a)** C contents is 3.3 wt%, **(b)** C contents is 3.6 wt%,** (c)** Si contents is 1.9 wt%,** (d)** Si contents is 2.3 wt%,** (e)** Mn contents is 0.1 wt%,** (f)** Mn contents is 0.7 wt%,** (g)** Cooling time (min) 145, **(h)** Cooling time (min) 265.

Figure 2 is the fracture morphology of fracture toughness test of the measured data in this paper (part of the measured data is selected), the composition of each sample is the same as Fig. 1.

It can be seen from Fig. 2a,b that an appropriate increase in C content can increase the number of graphite spheres, and a large number of graphite spheres play a role in releasing stress concentration when subjected to external forces, and the heavy-section ductile iron has more dimples and is more inclined to ductile fracture. It can be seen from Fig. 2c,d that when the Si content reaches 2.3 wt%, the increase in the number of graphite spheres and the increase in roundness also lead to the development of the heavy-section ductile iron from ductile–brittle mixed fracture to ductile fracture. The increase of Mn content leads to a significant increase in the number of pearlite in the matrix structure, and a large amount of chunky graphite is produced, which leads to a large number of cleavage planes and river patterns on the fracture surface, and the fracture morphology changes from ductile–brittle mixed fracture to brittle fracture, As shown in Fig. 2e,f. When the cooling rate of heavy-section ductile iron is accelerated, a large number of small and round graphite balls make the fracture morphology show a typical ductile fracture, As shown in Fig. 2g,h.

**Figure 3:**
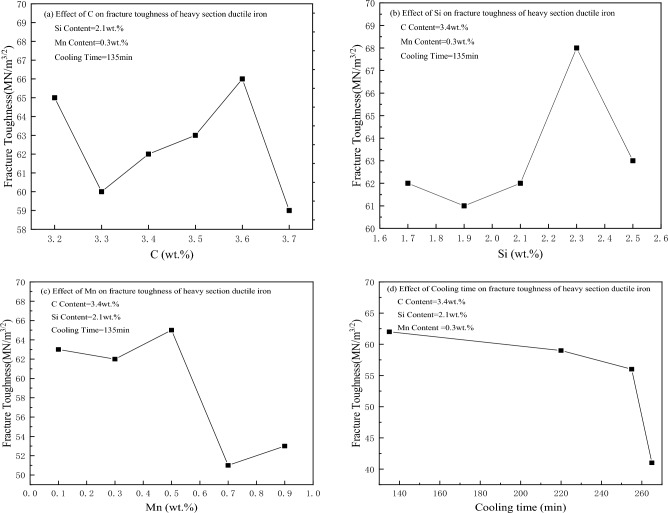
Effect of C, Si, Mn and cooling time on the fracture toughness of heavy section ductile iron (part of the measured data).

Figure 3 shows the measured data in this paper. The influence of C, Si, Mn and cooling speed (selected part of the measured data) on the fracture toughness of heavy-section ductile iron was investigated. Figure 3 shows that the chemical elements C, Si, Mn on the fracture toughness of heavy-section ductile iron present a nonlinear relationship, and only the cooling rate on the fracture toughness effect shows a linear relationship. With increasing cooling rate, the fracture toughness increases. This leads to the preparation of high fracture toughness heavy-section ductile iron parts in actual production, and a large number of tests need to be carried out to explore the combination of a reasonable chemical composition and forced cooling method.

## Methods

### Test sample preparation

In this paper, a total of 18 large heavy-section ductile iron test blocks with different chemical compositions were cast. Benxi Q12 pig iron, 75% silicon iron, steel grade 45 and graphite powder were melted in a medium-frequency induction furnace, and a spheroidizing treatment was applied using a Ce–Mg–Si nodulizing agent. The molten metal was poured into sand using furan resin sand to obtain heavy-section ductile iron cubic test blocks with a size of 400 mm × 400 mm × 400 mm. The chemical composition of nodulizing agent is shown in Table 1, and 18 heavy-section ductile iron test blocks with different C, Si, and Mn contents were labeled Casting 1, Casting 2, Casting 3, and Casting 18, and their chemical compositions are listed in Table 2.

**Table 1:** Composition of the nodulizing agent (wt%).

Elements of nodularizer	Ce	Mg	Si	Mn	Ca	Ti
Content	6.49	7.88	43.04	2.0	< 3	1.0

**Table 2:** Compositions of the castings (wt%).

Elements	C	Si	Mn	S	P	Mg
Casting1	3.2	1.70	0.1	< 0.02	< 0.05	< 0.04
Casting2	3.3	1.9	0.1	< 0.02	< 0.05	< 0.04
Casting3	3.4	2.1	0.1	< 0.02	< 0.05	< 0.04
Casting4	3.5	2.3	0.1	< 0.02	< 0.05	< 0.04
Casting5	3.6	2.5	0.1	< 0.02	< 0.05	< 0.04
Casting6	3.7	2.7	0.1	< 0.02	< 0.05	< 0.04
Casting7	3.2	1.7	0.3	< 0.02	< 0.05	< 0.04
Casting8	3.4	2.1	0.3	< 0.02	< 0.05	< 0.04
Casting9	3.6	2.5	0.3	< 0.02	< 0.05	< 0.04
Casting10	3.3	1.7	0.5	< 0.02	< 0.05	< 0.04
Casting11	3.5	2.1	0.5	< 0.02	< 0.05	< 0.04
Casting12	3.7	2.5	0.5	< 0.02	< 0.05	< 0.04
Casting13	3.2	1.7	0.7	< 0.02	< 0.05	< 0.04
Casting14	3.4	2.1	0.7	< 0.02	< 0.05	< 0.04
Casting15	3.6	2.5	0.7	< 0.02	< 0.05	< 0.04
Casting16	3.3	1.7	0.9	< 0.02	< 0.05	< 0.04
Casting17	3.5	2.1	0.9	< 0.02	< 0.05	< 0.04
Casting18	3.7	2.5	0.9	< 0.02	< 0.05	< 0.04

**Figure 4:**
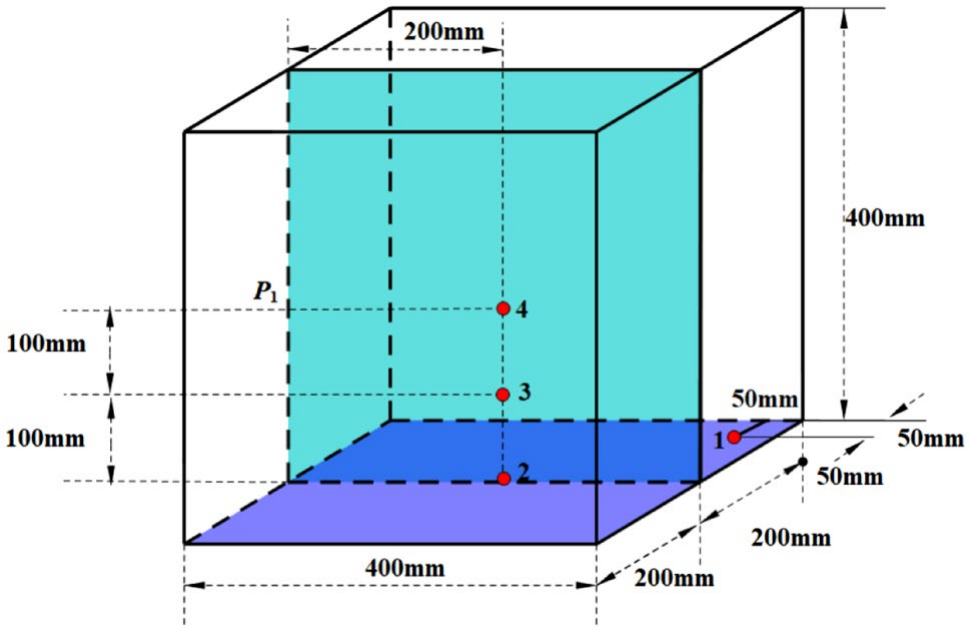
Four positions in castings chosen for temperature measurement and specimen collection.

**Figure 5:**
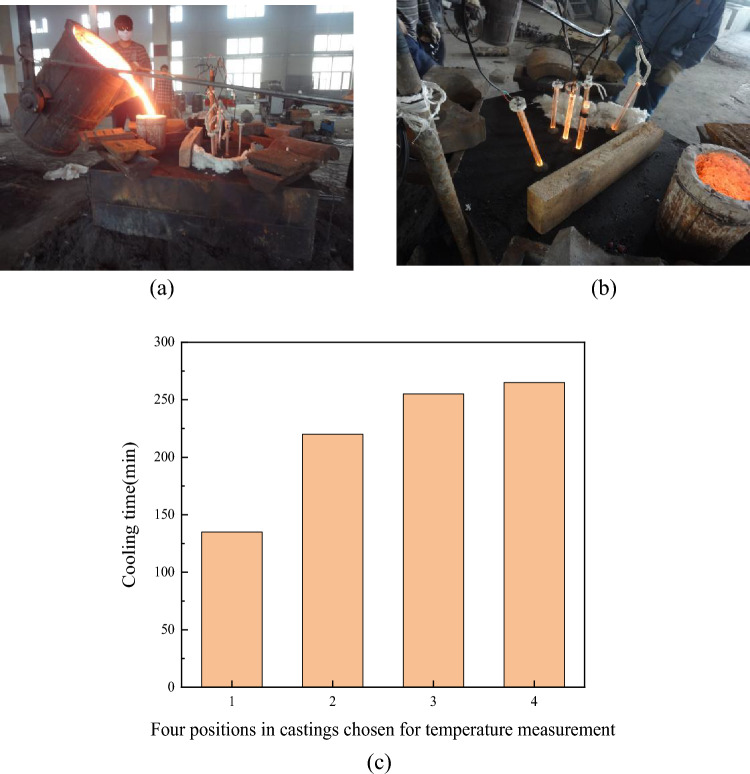
Temperature measurement process of pouring and the cooling time of the four positions in castings (**a**) Casting process, (**b**) temperature measurement process, (**c**) cooling time of four positions in castings.

Four positions were selected from the edge to the heart of the 18 test blocks as the typical cooling rate site sampling of heavy-section ductile iron, as shown in Fig. 4. A total of 72 specimens were sampled for microstructure observation and fracture toughness measurement. The cooling conditions of the 18 test blocks were the same, and the temperature was measured by using platinum–rhodium thermocouples and the configuration king temperature measurement system. Figure 5 shows the casting temperature measurement process and the solidification time of the 4 temperature measurement positions. From Fig. 5c, it can be seen that position 1 has the fastest cooling speed, with a solidification time of 135 min; followed by position 2, with a solidification time of 220 min; and position 3 and position 4, with solidification times of 255 and 265 min, respectively, which each exceed more than 250 min.

In this paper, the fracture toughness prediction of heavy-section ductile iron was investigated, the chemical elements C, Si, Mn and cooling time were the four factors used as input data for machine learning, and the fracture toughness was used as the output data to establish a machine learning prediction model. The total number of samples was 72, and all sample data were measured data.

### Fracture toughness test of heavy-section ductile iron

**Figure 6:**
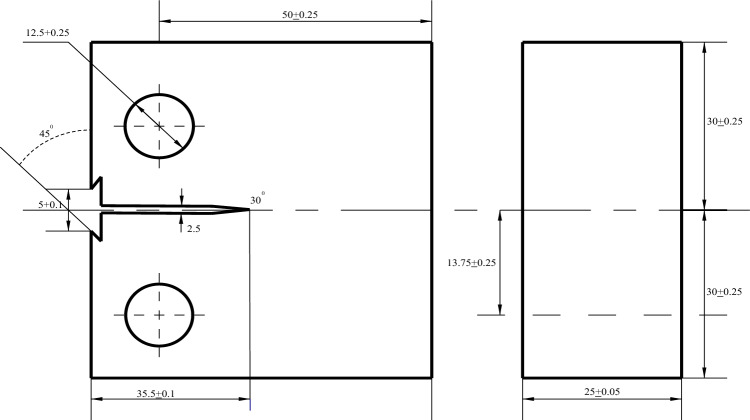
Dimensions of the fracture toughness specimen in mm.

The fracture toughness was tested at room temperature using an MTS 809 electrohydraulic servo material testing machine according to the standard GB/T4161-2007. The dimensions of the fracture toughness compact tensile (CT) specimens are shown in Fig. 6.

In this paper, six machine learning methods were selected to study the influence of C, Si, Mn and the cooling rate on the fracture toughness of heavy-section ductile iron, and corresponding machine learning model of regression prediction of fracture toughness of heavy-section ductile iron was constructed.

### XGBoost model

XGBoost is a parallel regression tree model based on the idea of boosting. Boosting refers to the weighted summation of a number of existing classifiers to obtain the final classifier. The XGBoost model was improved on the basis of the gradient descent decision tree (GBDT) by Chen^30^. In this model, each nonleaf node represents a feature, and leaf nodes represent a kind of label or decision result. When applying the decision tree, the samples to be predicted are examined for judgment conditions according to the feature values corresponding to the nodes to determine the next node position and judgment conditions and iteratively until a definite decision result is obtained.

Since the decision tree structure is simple and logical, it is prone to overfitting. Therefore, a random forest approach to reduce overfitting has been derived. The random forest constructs multiple random training sets and trains a weak learner decision tree based on them. Then, the decision trees are integrated to obtain a strong learner decision tree with superior performance to avoid overfitting^31^. Since the integrated decision trees are independent of each other and without feedback, a gradient descent decision tree method was derived.

Each tree in the GBDT (gradient-boosted decision tree) is fitted using the residuals (i.e., error-free observations) of the previous tree, and the final result is determined by the sum of the results of all trees. However, this also makes it impossible to perform parallel operations. XGBoost is further extended based on the GBDT method by presorting and saving the training data before the model is trained and using the sorted data in the iteration process^32^. This model calculates the feature gain at the time of new node selection and selects the node with the larger value to split and form the next layer of child nodes. Due to the use of prearranged data, XGBoost can split multiple features at the same time, thus realizing parallel operation and saving model training time. At the same time, its objective function not only includes the common loss function but also adds a regularization term and uses the column method in random forest, which reduces overfitting and accelerates the speed of parallel computation. The most important core issue of the XGBoost algorithm is the objective function that combines the evaluation error of decision trees:

1 $$ predict(x) = \sum\limits_{j}^{n} {\left( {Loss\gamma_{j} ,\hat{y}_{j} } \right)} + \sum\limits_{m = 1}^{M} {\gamma \left( {f_{m} } \right)} . $$

In the equation, Loss is the loss function of the deviation between the actual value and the predicted value, $y_{j}$ is the actual value of the predicted data, $\hat{y}_{i}$ is the result of the previous decision tree algorithm, x is the input data, thus fitting the algorithm results of multiple decision trees, while $f_{m}$ is the approximation function used by the decision tree. Meanwhile, $\gamma$ is the regularization term for improving the penalty coefficient used by the decision tree, and the basic decision tree approximation function is:

2 $$ y_{i} = \sum\limits_{m = 1}^{M} {f_{m} \left( x \right)} ,\quad f_{m} \in F. $$

**Figure 7:**
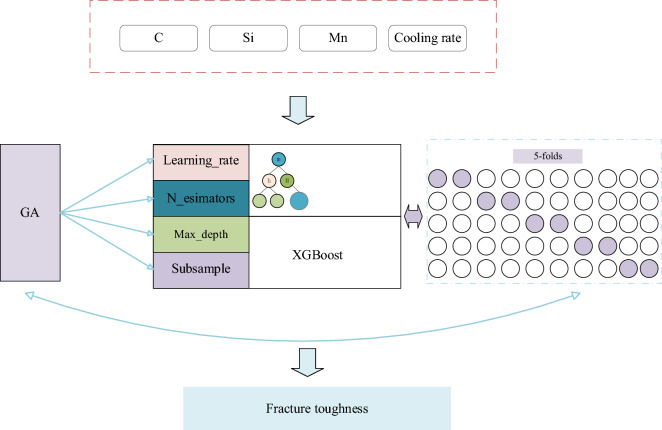
XGBoost model structure.

In this paper, the XGBoost model parameters are searched by the random search method, and fivefold cross validation is used to evaluate the model performance to finally arrive at the best model. The model graph is shown in Fig. 7.

### Support vector regression

The regression algorithm is support vector regression or SVR. SVR is a supervised learning algorithm used to predict discrete values. Support vector regression uses the same principles as those in SVM. The basic idea is to map the data into a high-dimensional space and find the optimal hyperplane for regression and thus the line of best fit. Compared with traditional regression algorithms, SVR not only considers the degree of data fit but also the generalizability of the model. Thus, SVR can effectively deal with high-dimensional data and nonlinear data^33^. In the regression task, it is necessary to make the interval between the sample points that are farthest away from each other by the hyperplane the largest. That is, the SVR is given a restriction on the interval, and the deviation of the regression model f (x) from y must be $\le \varepsilon$ for all sample points. This range is referred to as the deviation of the *ε* pipeline.

The SVR optimization problem can be expressed mathematically as^34^:

3 $$ \mathop {\min }\limits_{w,b} \frac{1}{2}\left\| w \right\|_{2}^{2} , $$

4 $$ s.t\;\left| {y_{i} - \left( {\sigma^{T} x_{i} + b} \right)} \right| \le \varepsilon ,\;i = 1,2, \ldots ,N. $$

**Figure 8:**
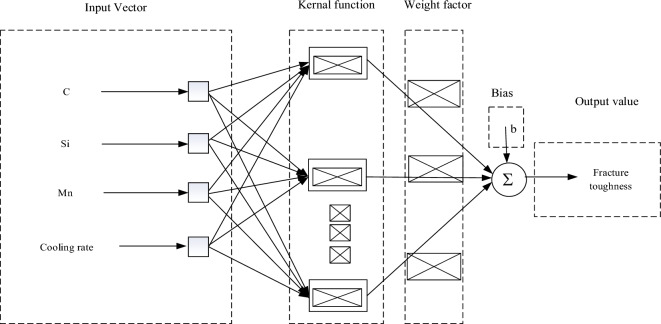
Structure of the SVR model.

Given samples $D = \{ (x_{1} ,y_{1} )(x_{2} ,y_{2} ),...,(x_{n} ,y_{n} )\} ,{\text{y}}i \in R$, f (x) is obtained such that it is as close as possible to y, and w and b are the parameters to be determined. The loss is zero when f (x) and y are identical. Support vector regression assumes that there is at most ε deviation between f (x) that can be tolerated. The loss is calculated when and only when the absolute value of the difference between f (x) and y is greater than ε, which is equivalent to the construction of a spacing band with a width of 2^ε^ centered around f (x). The training samples are considered to be correctly predicted if they fall into this spacing band, as shown in Fig. 8.

### Multi-layer perception model

**Figure 9:**
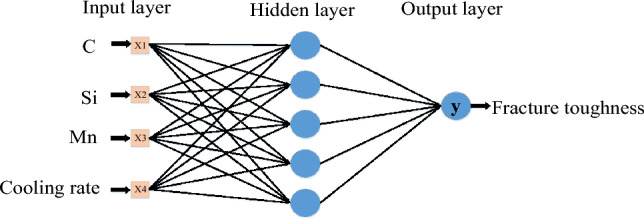
MLP model diagram.

The MLP regressor is a supervised learning algorithm. Figure 9 shows the MLP model with only 1 hidden layer; the left side is the input layer, and the right side is the output layer^35^.

The MLP is also known as the multilayer perceptron. In addition to the input and output layers, it can have more than one hidden layer in the middle. A linearly divisible data problem can be solved if there is no hidden layer^36^. The layers of the multilayer perceptron shown in Fig. 9 are fully connected to each other. Therefore, it is possible to solve the problem without any hidden layer. The bottom layer of the multilayer perceptron is the input layer, the middle layer is the hidden layer, and the last layer is the output layer. The input layer is a 4-dimensional vector, and the output is 4 neurons^37^. The neurons in the hidden layer are fully connected to the input layer. It is assumed that the input layer is represented by the vector Xi, and the output of the hidden layer is f (W_kj_X + b1), Wji is the weight (also known as the connection coefficient), b1 is the bias (in the design model in Fig. 9, the bias is 0 by default).The function f can be the commonly used sigmoid function or tanh function. Finally, the output layer is connected to the hidden layer through a sigmoid function or tanh function. This connection is analogous to a multicategory logistic regression, that is, softmax regression. Therefore, the output of the output layer is y = softmax (W_kj_Xj + b2), where X_j_ represents the hidden layer output f (W_kj_Xj + b2), and b2 is the bias.

### Gaussian process regression model

The Gaussian process model is a statistical tool for constructing stochastic processes and is widely used in the fields of machine learning, statistics and information processing. The Gaussian process model is based on the principles of probability theory. By modeling and predicting observed data, this model can provide estimates of the probability distribution of unknown data points^38^.

In the Gaussian process model, it is assumed that the stochastic process under study obeys a multivariate Gaussian distribution for any set of inputs. Each point in the input space is usually projected to a random variable in the output space, and the joint assignment of these random variables constitutes the probabilistic model of the Gaussian process^39^.

A Gaussian process model can be described by two underlying components: the mean value function and the variance function (also known as the kernel function). The mean value function represents modeling the overall dynamics of the stochastic overprocess, while the covariance function describes the correlation or similarity between different points^40^.

Given a set of input points and corresponding observations, predictions can be made using a Gaussian process model. The result of the prediction is a conditional probability distribution over the unknown data points, which includes a predicted mean and a predicted uncertainty (variance). This estimate of uncertainty is quantified by the covariance function, which portrays the correlation between the input points, reflecting the uncertainty of the expected measurements^15^. Its mathematical expression is as follows:

5 $$ f(x) \sim GP(m(x),k(x,x^{\prime})). $$

Here, x is the mean, and y is the variance. The expression is as follows:

6 $$ m(x) = E[f(x)], $$

7 $$ k({\varvec{x}},\user2{x^{\prime}}) = E\{ [f({\varvec{x}}) - m({\varvec{x}})][f(\user2{x^{\prime}}) - m(\user2{x^{\prime}})]\} , $$

Considering that the general observations of the function are noisy, there is:

8 $$ y = f(x) + \varepsilon , $$

where $\varepsilon \sim N(0,\sigma_{n}^{2} )$ is the white noise with a variance of $\sigma_{n}^{2}$. The prior distribution of y can be expressed as:

9 $$ y \sim N\left( {0,K_{f} (x,x) + \sigma_{n}^{2} I_{n} } \right). $$

At the test dataset ${\varvec{x}}^{ * }$, the joint prior distribution of the observation set ***y*** and the prediction set ***y*^** can be expressed as:

10 $$ \left. {\left[ {\begin{array}{*{20}c} y \\ {\hat{y}} \\ \end{array} } \right] \sim {\text{N}}\left( {0,\left[ {\begin{array}{*{20}c} {K_{f} (x,x) + \sigma_{n}^{2} {\varvec{I}}} & {{\varvec{K}}_{f} ({\varvec{x}},{\varvec{x}}^{ * } )} \\ {{\varvec{K}}_{f} ({\varvec{x}},{\varvec{x}}^{ * } )^{{\text{T}}} } & {{\varvec{K}}_{f} ({\varvec{x}}^{ * } ,{\varvec{x}}^{ * } )} \\ \end{array} } \right]} \right.} \right). $$

According to the above equation, the posterior distribution ${\text{p}}({\hat{\mathbf{y}}}|{\varvec{x}},{\varvec{y}},{\varvec{x}}^{ * } )$ can be obtained:

11 $$ {\text{p}}({\hat{\mathbf{y}}}|{\varvec{x}},{\varvec{y}},{\varvec{x}}^{ * } ) = {\text{N}}({\hat{\mathbf{y}}}|{\hat{\mathbf{y}}}^{ * } ,{\text{cov}}({\hat{\mathbf{y}}}^{ * } )), $$

where the output of the model prediction is the predicted mean ${\hat{\mathbf{y}}}^{ * }$, and the uncertainty of the prediction model is reflected by the prediction covariance ${\text{cov}}(\hat{y}^{ * } )$. The expression is as follows:

12 $$ {\text{cov}}(\hat{y}^{ * } ) = K_{f} (x^{ * } ,x^{ * } ) - K_{f} (x,x^{ * } )^{{\text{T}}} [K_{f} (x,x) + \sigma_{n}^{2} {\varvec{I}}_{n} ]^{ - 1} {\varvec{K}}_{f} ({\varvec{x}},{\varvec{x}}^{ * } ). $$

**Figure 10:**
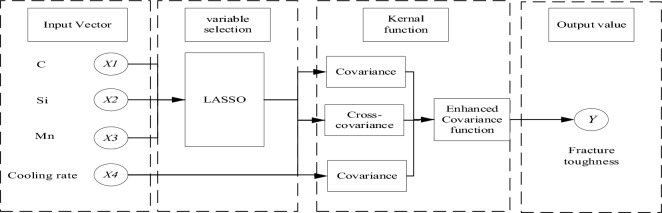
Diagram of the standard Gaussian process model.

The standard Gaussian process model can be represented as shown in Fig. 10.

### Bagging model

**Figure 11:**
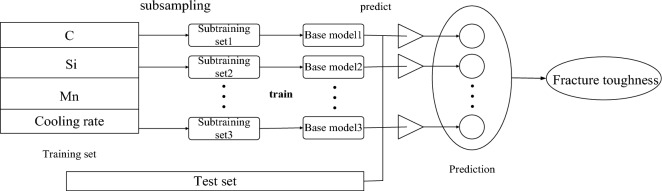
Schematic diagram of the bagging algorithm.

Bagging regression is an integrated learning method that generates multiple subsets of training data by randomly sampling the training data with replacement and then uses these subsets to train multiple base learning devices^41,42^. The main idea of bagging regression is to improve the generalizability of the model by reducing the variance. The specific process is to generate multiple subsets from the training set X by random sampling in a relaxed manner and use each subset to train a base learner. Each base learner is a regression model, such as decision tree regression or linear regression^43^. The main idea of bagging regression is to improve the generalizability of the model by reducing the variance. After training, the prediction results of the base learners are averaged or weighted average, and the final prediction results are obtained Y. Bagging regression reduces the variance of the model and improves the generalizability of the model. Parallelization is possible at the same time, accelerating model training. The process is shown in Fig. 11.

Let the expectation of the single model be $\mu$; then, the expectation of the bagging regression is:

13 $$ E\left( {\frac{1}{n}\sum\limits_{i = 1}^{n} {X_{i} } } \right) = \frac{1}{n}E\left( {\sum\limits_{i = 1}^{n} {X_{i} } } \right) = E(X_{i} ) \approx \mu . $$

n is the number of base learners, $X_{i}$ is the expectation of the i base learner. This expectation value represents the expected performance of the Bagging regression model on the entire training dataset, helping us evaluate the overall performance of the Bagging regression model.

### Random forest regression model

**Figure 12:**
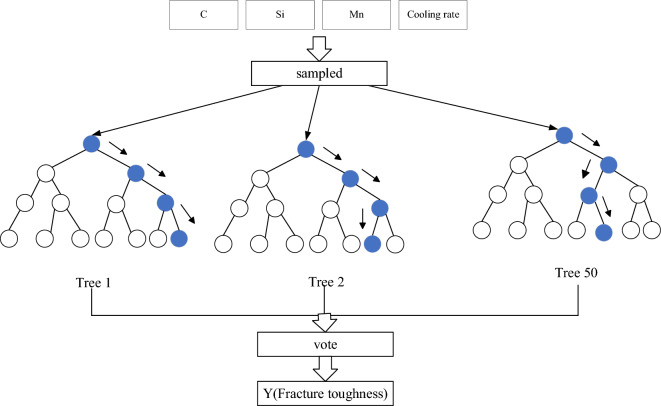
Random forest model structure.

The random forest is a more optimized combinatorial forecasting model proposed by professor Breiman in 2001 based on the idea of bagging, which is a new extension of randomness^44,45^. The random forest algorithm utilizes bootstrap sampling to construct different tree models with random samples. Then, the selection of the best node of each tree model is changed so that the variable node of each tree also has randomness, which in turn generates a number of regression trees with high prediction accuracy and uncorrelated regression trees^46^. The predictions of all the trees are average to vote, representing the prediction of this regression model. The RF model structure is shown in Fig. 12.

Let {$\left\{ {h\left( {x,\theta_{t} } \right)} \right\}$, t = 1,2,…,T} be a T regression tree in a random forest, where $\theta_{{\text{t}}}$ is a random variable obeying an independent homogeneous distribution and x is a dependent variable. Then, the regression result can be expressed as:

14 $$ \overline{h} \left( {\text{x}} \right) = \frac{1}{{\text{T}}}\sum\limits_{{{\text{t}} = 1}}^{{\text{T}}} {\{ h(x,\theta_{t} )\} } . $$

### Experimental conditions and settings (experiments)

The model is trained on measured fracture toughness data of large ductile cast iron. The hardware environment of the simulation platform for this experiment is a CPU and a GPU. The CPU is an Intel (R) Core i7-10700 @ 2.90 GHz, and the GPU is an NVIDIA GeForce GTX 1660 SUPER with 64 GB of RAM. The simulation software environment uses PyCharm, Python version 3.8, and the Sklearn, NumPy, pandas and matplotlib libraries.

In this paper, two indicators, the RMSE and *R*^2^, are selected to measure the reliability and accuracy of the model prediction. The RMSE metric measures the error of the prediction value deviating from the true value, and a smaller value represents a higher prediction accuracy. The *R*^*2*^ measures the ability of the model to fit the data. The closer to 1 this value is, the better the model fits, and the closer the 0 this value is, the worse the model fits. The formulas for RMSE and *R*^*2*^ are shown in Eqs. (15) and (16).

15 $$ RMSE = \frac{1}{N}\sum\limits_{t = 1}^{N} {\left( {y_{true}^{t} - y_{pred}^{t} } \right)} , $$

16 $$ R^{2} = 1 - \frac{{\sum\limits_{t = 1}^{N} {\left( {y_{true}^{t} - y_{pred}^{t} } \right)^{2} } }}{{\sum\limits_{t = 1}^{N} {\left( {y_{true}^{t} - y_{avg}^{t} } \right)^{2} } }}. $$

Among them, the *R*^2^ value is in the range of (0, 1). The closer to 1 the value is, the better the prediction result. In contrast, the closer to 0 the value is, the worse the prediction result. The RMSE value is in the range of (zero, + ∞). A value closer to 0 indicates a smaller prediction error.

Genetic algorithm is a heuristic search and optimization technique inspired by the processes of biological evolution in nature. It simulates the processes of biological evolution, such as selection, crossover, and mutation, to find the optimal solution or better solution to the problem. It is widely used in the field of machine learning. In this study, the samples are trained using the fifty percent cross-validation method, and the hyperparameter optimization method is the genetic algorithm (GA). XGBoost has numerous hyperparameters that need to be manually set. In this paper, we selected several common hyperparameters: n_estimators (number of trees), max_depth (maximum tree depth), learning rate, and subsample. For the SVR model, we chose C (penalty parameter), gamma (kernel coefficient), and kernel type. The MLP Regressor model selected alpha (regularization parameter), max_iter (maximum number of iterations), and solver for optimizer selection. Gaussian Process Regression model parameters included alpha (value added to the diagonal of the kernel matrix during model fitting) and kernel type. For the Bagging model, we chose n_estimators (number of base estimators), max_samples (number of samples to train base estimators), max_features (number of features to train base estimators), and bootstrap (determines the sampling method for the sample subset). The Random Forest model selected n_estimators (number of trees) and maxdepth (maximum tree depth). The specific model coefficients and optimized coefficients are shown in Table 3.

**Table 3:** Parameter ranges and optimal parameters for each model.

Model	Parameters and scope	GA optimized parameters
XGBoost	max_depth: [1, 10] learning_rate: [0.01, 0.3] n_estimators: [50, 200] subsample: [0.5, 1]	max_depth = 4 learning_rate = 0.2 n_estimators = 82 subsample = 0.87
SVR	C: [0.1, 1, 10, 100] gamma: [0.1, 1, 10] kernel: [rbf, linear, relu]	C = 10 gamma = 0.1 kernel: ‘rbf’
MLP regressor	alpha: [0.0001, 0.001, 0.01, 1] max_iter: [100, 200, 300, 400, 500, 600, 700, 800, 900, 1000] solver: [‘adam’, ’lbfgs’]	hidden_layer_sizes = (16) solver = ‘lbfgs’ alpha = 0.001 max_iter = 600
Gaussian process regression	alpha: np.logspace (− 5, 0, 6), kernel: [RBF, Matern]	alpha = 0.1 kernel: Matern (length_scale = 1, nu = 0.5)
Bagging	n_estimators: [10, 50, 100] max_samples: [0.5, 1.0] max_features: [0.5, 1.0] bootstrap: [True, False]	n_estimators = 100 max_samples = 1.0 max_features = 1.0 bootstrap: True
Random forest	n_estimators; [50, 100, 150, 200] maxdepth: [3, 5, 7]	n_estimators = 50 maxdepth = 7

## Results and analysis

**Figure 13:**
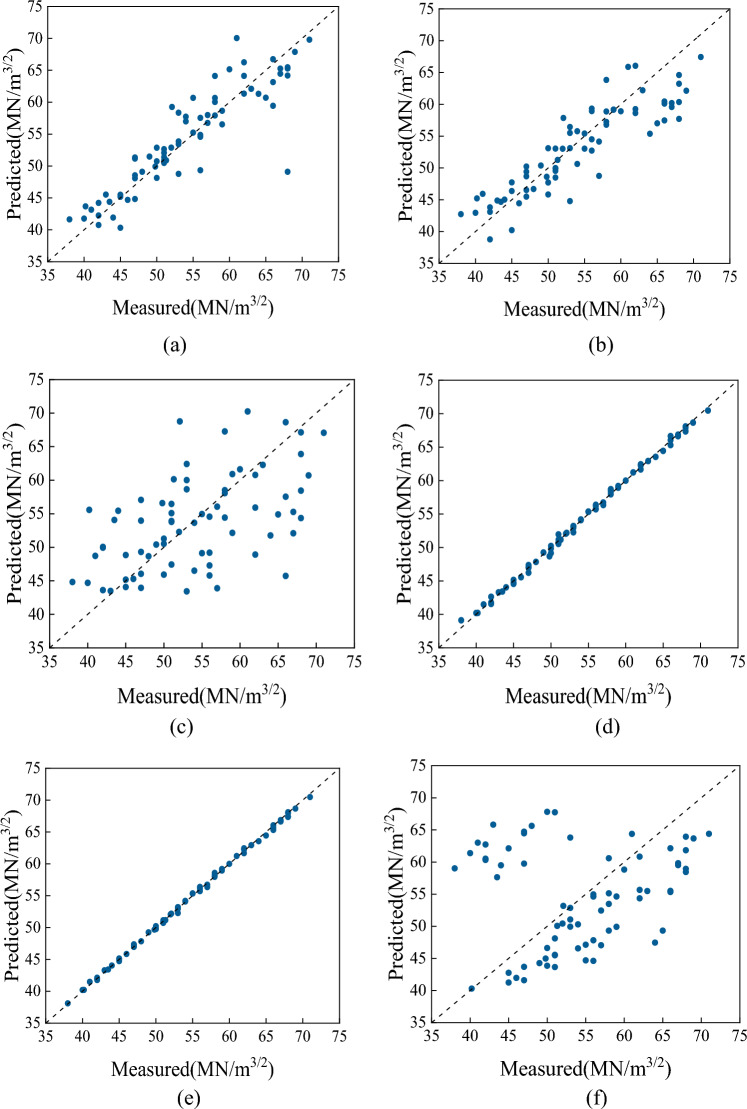
Comparison of the degree of fit of the models (**a**) XGBoost, (**b**) SVR, (**c**) MLP, (**d**) Gaussian process regression, (**e**) Bagging, (**f**) Random forest.

Figure 13 illustrates the comparison of the goodness of fit of six machine learning models optimized through genetic algorithms. As shown in Fig. 13, the optimized XGBoost, SVR, MLP, and Random Forest models exhibit poor fitting of predicted values to actual values, while the optimized Bagging and Gaussian process models show a good fit between predicted and actual values, approaching a single line.

**Figure 14:**
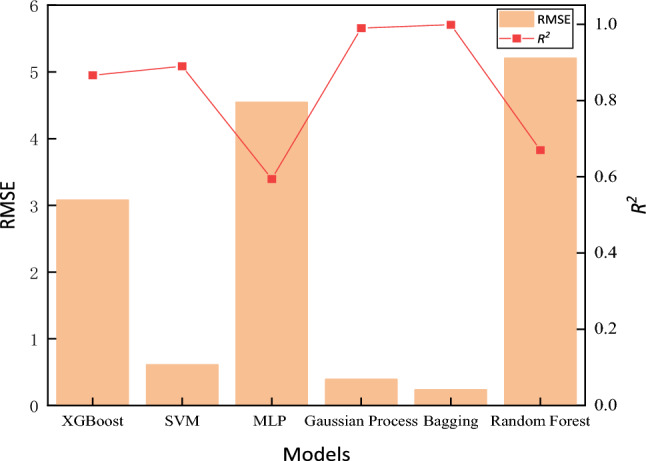
Evaluation indicators for each model.

To further compare the accuracy of the Gaussian process and bagging models in predicting the fracture toughness of heavy-section ductile iron, the RMSE and *R*^2^ are selected as the two metrics to measure the reliability and accuracy of the model predictions. As shown in Fig. 14, the *R*^2^ values of the XGBoost, SVR, MLP regressor, Gaussian process and random forest models are 0.8662, 0.8901, 0.5942, 0.99 and 0.63, respectively, which are lower than the* R*^2^ value of bagging (0.9990). The RMSEs of the XGBoost, SVR, MLP regressor, Gaussian process and random forest models are 3.085, 0.661, 4.5467, 0.3937 and 5.21, respectively, which are higher than the RMSE of 0.2373 of the bagging model. Therefore, the bagging model is better for predicting the fracture toughness of heavy-section ductile iron.

**Figure 15:**
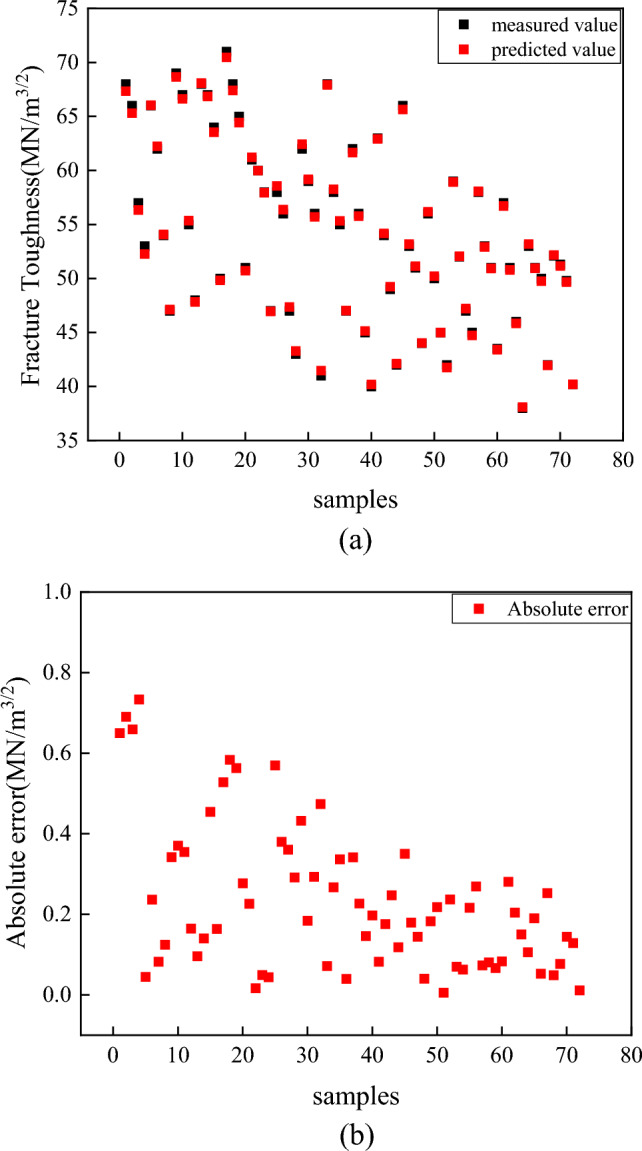
Bagging model prediction results (**a**) Comparison of the true and predicted values, (**b**) absolute error.

The bagging model optimized by the genetic algorithm is applied to 72 fracture toughness specimens of heavy-section ductile iron with different compositions and cooling times constructed in this paper. The experimental results are shown in Fig. 15a. The results indicate that the projected value and the true value data points basically coincide with each other, and the prediction effect is accurate. Figure 15b shows the absolute value of the prediction error. The figure shows that the prediction error is basically less than 0.6, and the maximum does not exceed 0.8.

Bagging is an ensemble learning method that works by constructing multiple weak learners (typically decision trees) and combining their results to improve the overall model performance. On the other hand, genetic algorithm is an optimization algorithm that simulates the biological evolution process, searching for the optimal solution to a problem by mimicking evolutionary mechanisms such as natural selection, crossover, and mutation. Through genetic algorithm optimization, the Bagging model can better adapt to different data patterns, improve prediction accuracy for unknown data, and particularly demonstrate significant advantages for materials with complex structures such as thick-sectioned ductile cast iron.

In this paper, six machine-learning models optimized by the genetic algorithm are applied to 72 heavy-section ductile iron specimens with different compositions and cooling times. The experimental results show that the optimized bagging model has the best prediction effect, with an *R*^2^ of 0.9990 and an RMSE of 0.2373.

The general C content of heavy-section ductile iron is controlled between 3.5 and 3.9 wt%, appropriate carbon content can make cast iron have good fluidity and lubricity, which is convenient for filling mold cavity. However, if the C content is too high to exceed 3.9 wt%, the plasticity and toughness of heavy-section ductile iron will be seriously reduced, and the ductile iron is prone to crack and fracture, the thermal brittleness increases^47^. The general Si content of heavy-section ductile iron is controlled between 1.7 and 3.8 wt%, the hardness, tensile strength and yield strength of heavy-section ductile iron can be improved by adding appropriate amount of silicon. However, the ductile–brittle transition temperature of ductile iron can be significantly increased and the plastic and toughness of heavy-section ductile iron will be reduced when the silicon content is too high to exceed 3.8 wt%^48^. The preparation of heavy-section ductile iron generally requires its ferrite matrix to obtain high fracture toughness. Due to the long cooling time in the core of heavy-section ductile iron, it is easy to generate a large amount of pearlite, and its Mn content needs to be strictly controlled, generally controlled within 0.5 wt%^49^.

In this paper, in order to explore the performance prediction of fracture toughness of heavy-section ductile iron products, the content range of C, Si and Mn of heavy-section ductile iron is expanded in the additional test, as shown in Table 4.

**Table 4:** Compositions of the additional castings (wt%).

Elements	C	Si	Mn	S	P	Mg
Casting19	4.0	3.0	1.0	< 0.02	< 0.05	< 0.04
Casting20	4.0	3.4	1.2	< 0.02	< 0.05	< 0.04
Casting21	4.2	3.8	1.4	< 0.02	< 0.05	< 0.04
Casting22	4.2	4.0	1.6	< 0.02	< 0.05	< 0.04

As shown in Table 4, additional experiments were conducted to prepare four heavy-section ductile iron specimens with varying carbon (C) content, silicon (Si) content, and manganese (Mn) content. A total of 16 samples were cut from the edge to the core of each specimen, and the cooling time was the same as that of the previous samples. The basic parameters for each model were the same as those in Table 3, and the same methods were employed.

**Figure 16:**
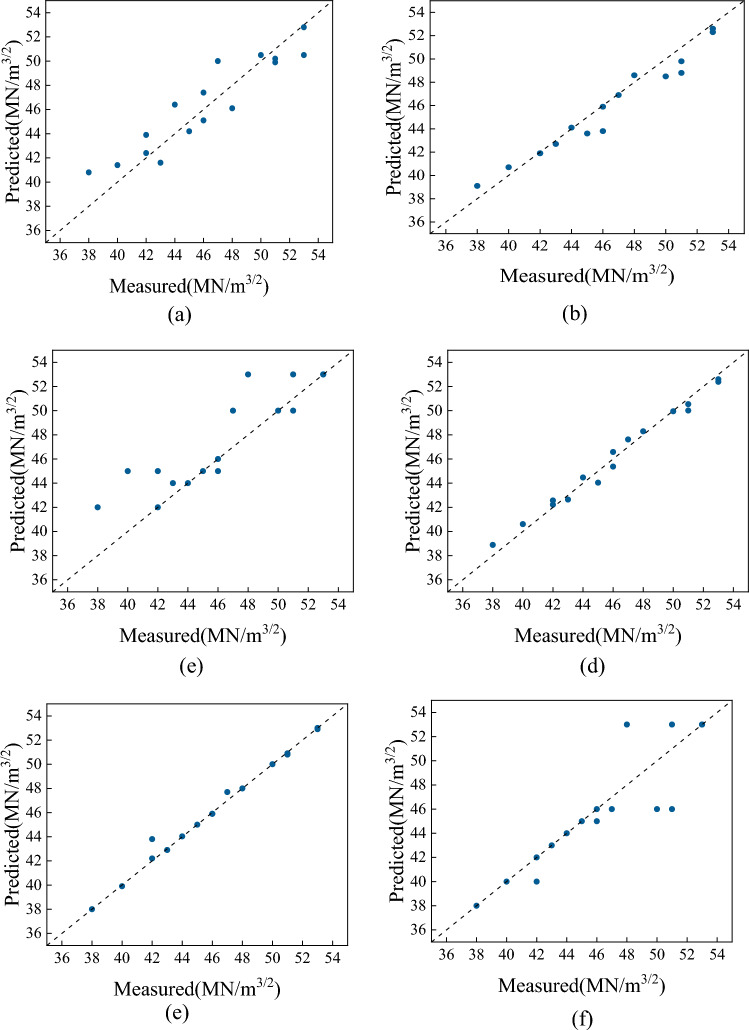
Comparison of the fitting degree of each model. (**a**) XGBoost, (**b**) SVR, (**c**) MLP, (**d**) Gaussian process regression, (**e**) Bagging, (**f**) Random forest.

Figure 16 compares the fitting degree of six machine learning models. From the figure, it can be seen that the fitting effect of the Bagging model optimized by genetic algorithm and the Gaussian process regression model is still better, approaching a straight line.

**Figure 17:**
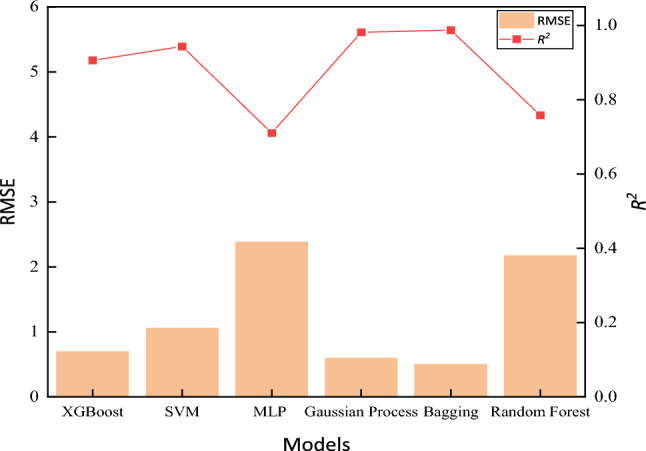
Evaluation indicators for each model.

To further compare the accuracy of Gaussian Process and Bagging models in predicting the fracture toughness of heavy-section ductile iron, this study selected the root mean square error (RMSE) and coefficient of determination (*R*^2^) as two indicators to measure the reliability and accuracy of the model predictions. As shown in Fig. 17, the *R*^2^ values for XGBoost, SVR, MLP Regressor, Gaussian Process, and Random Forest models are 0.9061, 0.9435, 0.7105, 0.9818, and 0.7582, respectively, all lower than Bagging 0.9873. Additionally, the RMSE values for these models are 0.6936, 1.0537, 2.38, 0.5938, and 2.17, all higher than Bagging’s 0.4993. The results indicate that after expanding the component range of heavy-section ductile iron products, the obtained results still meet the requirements for practical applications.

## Conclusions

1. In this paper, four factors affecting the fracture toughness of heavy-section ductile iron, such as C content, Si content, Mn content and cooling rate, are discussed. By using the isothermal section method, 18 cubic physical simulation specimens with different compositions and wall thicknesses of 400 mm were cast, and 72 specimens were prepared for microstructure observation and fracture toughness testing. In addition, the relationship between the above four factors and the microstructure and fracture toughness of heavy-section ductile iron was discussed.
2. Aiming at the problems of a long preparation cycle, high R&D cost, and many nonlinear influences for high fracture toughness heavy-section ductile iron, a machine-learning-based fracture toughness prediction model for thick and large section ductile iron was established. The C content, Si content, Mn content and cooling rate are input, and the fracture toughness is output.
3. Compared with the XGBoost, SVM, MLP regressor, Gaussian process, random forest and other models, the bagging model has the best prediction effect, followed by the Gaussian process, with the *R*^2^ values reaching 0.9990 and 0.99, respectively, and RMSE values of 0.2373 and 0.3937, respectively. These models can meet the design requirements of high fracture toughness heavy-section ductile iron for nuclear spent fuel storage and transportation containers and bases of wind power.

## Data Availability

The datasets used and analyzed during the current study available from the corresponding author on reasonable request.
